# Tirzepatide is an imbalanced and biased dual GIP and GLP-1 receptor agonist

**DOI:** 10.1172/jci.insight.140532

**Published:** 2020-09-03

**Authors:** Francis S. Willard, Jonathan D. Douros, Maria B.N. Gabe, Aaron D. Showalter, David B. Wainscott, Todd M. Suter, Megan E. Capozzi, Wijnand J.C. van der Velden, Cynthia Stutsman, Guemalli R. Cardona, Shweta Urva, Paul J. Emmerson, Jens J. Holst, David A. D’Alessio, Matthew P. Coghlan, Mette M. Rosenkilde, Jonathan E. Campbell, Kyle W. Sloop

**Affiliations:** 1 Quantitative Biology, Lilly Research Laboratories, Eli Lilly and Company, Indianapolis, Indiana, USA.; 2 Duke Molecular Physiology Institute, Duke University, Durham, North Carolina, USA.; 3 Department of Biomedical Sciences and NNF Center for Basic Metabolic Research, University of Copenhagen, Copenhagen, Denmark.; 4 Diabetes and Complications, and; 5 PK/PD & Pharmacometrics, Lilly Research Laboratories, Eli Lilly and Company, Indianapolis, Indiana, USA.

**Keywords:** Therapeutics, Diabetes

## Abstract

Tirzepatide (LY3298176) is a dual GIP and GLP-1 receptor agonist under development for the treatment of type 2 diabetes mellitus (T2DM), obesity, and nonalcoholic steatohepatitis. Early phase trials in T2DM indicate that tirzepatide improves clinical outcomes beyond those achieved by a selective GLP-1 receptor agonist. Therefore, we hypothesized that the integrated potency and signaling properties of tirzepatide provide a unique pharmacological profile tailored for improving broad metabolic control. Here, we establish methodology for calculating occupancy of each receptor for clinically efficacious doses of the drug. This analysis reveals a greater degree of engagement of tirzepatide for the GIP receptor than the GLP-1 receptor, corroborating an imbalanced mechanism of action. Pharmacologically, signaling studies demonstrate that tirzepatide mimics the actions of native GIP at the GIP receptor but shows bias at the GLP-1 receptor to favor cAMP generation over β-arrestin recruitment, coincident with a weaker ability to drive GLP-1 receptor internalization compared with GLP-1. Experiments in primary islets reveal β-arrestin1 limits the insulin response to GLP-1, but not GIP or tirzepatide, suggesting that the biased agonism of tirzepatide enhances insulin secretion. Imbalance toward GIP receptor, combined with distinct signaling properties at the GLP-1 receptor, together may account for the promising efficacy of this investigational agent.

## Abstract 2

Studies of the dual GIP and GLP-1 receptor agonist tirzepatide reveal occupancy favoring the GIP receptor and biased cAMP signaling at the GLP-1 receptor.

## Introduction

Treatments that intervene at more than one regulatory pathway are used to combat a variety of complex diseases, including psychiatric disorders, infectious diseases, many cancers, hypertension, and metabolic conditions such as obesity and type 2 diabetes mellitus (T2DM) (1–6). These approaches are intended to enhance efficacy by synergistic responses that result from engaging complementary mechanisms and typically require poly-pharmacologic regimens where multiple drugs are administered. Peptide engineering is further advancing this concept by creating single agents possessing activity at more than one pharmacologic target. Many of these are designed to activate GPCRs that control glucose homeostasis and energy balance. Several of these multireceptor agonists have now entered clinical development for the treatment of T2DM, obesity, or nonalcoholic steatohepatitis (7, 8). The most advanced of these agents is the dual glucose-dependent insulinotropic polypeptide receptor (GIPR) and glucagon-like peptide-1 receptor (GLP-1R) agonist, tirzepatide (LY3298176).

The initial clinical investigation of tirzepatide shows unprecedented efficacy for glucose lowering and weight loss in T2DM as about 30% of patients receiving the 15 mg dose reached normoglycemia (HbA1C < 5.7% per the ADA definition) and 1 in 4 subjects lost ≥ 15% of their body weight in a 26-week phase 2b trial (9). In addition, tirzepatide treatment has shown a strong effect on lowering fasting concentrations of circulating triglycerides (9), and HOMA2-IR analysis points to an improvement in insulin sensitivity that is only partly accounted for by the weight loss (10). This broad improvement in metabolic health is in line with the hypothesis that adding GIP pharmacology to GLP-1 therapy improves glycemic control by dual actions on pancreatic β cells to enhance insulin secretion, GIP-driven improvements in white adipose tissue function, and a strong anorexigenic effect from integrating the activation signals of both receptor pathways in the brain (11). Importantly, while the actions of dual GIPR and GLP-1R agonism are tailored to improve glycemic control and reduce body weight, distinct pharmacological characteristics at both receptors may be central to tirzepatide achieving the degree of efficacy observed.

Tirzepatide was discovered by engineering GLP-1 activity into the GIP sequence (12). It is an imbalanced dual agonist in favor of GIPR over GLP-1R activity as the molecule shows equal affinity for the GIPR compared with native GIP but binds the GLP-1R with approximately 5-fold weaker affinity than native GLP-1. The imbalanced nature of tirzepatide may be critical to maximizing the efficacy of a dual agonist because dose escalation for GLP-1R activation can be limited by gastrointestinal effects such as nausea and vomiting (13), while GIPR engagement is not known to be associated with similar events. As a consequence, a pharmacological profile favoring potency at the GIPR offers an opportunity to fully engage this pathway while minimizing GLP-1–related tolerability issues. In addition to a potency ratio toward GIPR activity, tirzepatide contains a C20 unsaturated di-acid acyl chain to affect albumin binding, and the overall properties of the molecule enable once-weekly dosing (12). Beyond convenience, this pharmacokinetic feature may confer pharmacodynamic benefit, as high and sustained concentrations of selective GLP-1R agonists have enhanced clinical efficacy (14, 15).

Despite the compelling effects of tirzepatide on glucose and body weight regulation in preclinical and clinical studies (9, 12), there remain significant gaps in understanding its mechanism of action. In the study presented here, the activation of important signaling pathways downstream of the GIPR and GLP-1R by tirzepatide was investigated. These studies are supported by ex vivo experiments using pancreatic islets isolated from genetic mouse models. Furthermore, the influence of human albumin on the potency differential of tirzepatide for activating both the GIPR and GLP-1R is used to determine free drug concentrations, allowing estimates of receptor occupancy for tirzepatide at clinically efficacious drug levels. Together, these data point to a unique pharmacologic profile underlying the potent actions of this multireceptor agonist.

## Results

### Tirzepatide is an imbalanced agonist of the GIPR and GLP-1R and shows biased signaling at the GLP-1R.

A thorough understanding of the pharmacological properties of multireceptor agonists is essential for predicting the characteristics of drugs that are required for clinical efficacy. To date, the majority of such agents being developed to treat metabolic diseases possess activity at the GLP-1R as the anchor pharmacophore but are also agonists for either the GIPR, the glucagon receptor, or both (7, 8). Ultimately, an optimal mix of properties, including distinct receptor potencies and an ability to stimulate certain intracellular signaling pathways, along with a pharmacokinetic profile allowing desirable receptor occupancy, should advance efficacy beyond that observed for selective GLP-1R agonists. Since tirzepatide is the first multireceptor agonist to enter a phase 3 program, evaluating its pharmacological attributes in the context of the efficacy findings from the phase 2 studies establishes a benchmark for comparing future molecules of the class.

Characterizing the activation of GIPR and GLP-1R by tirzepatide and connecting these actions with biological activity is fundamental to understanding its pharmacology. The study of GPCRs is often confounded by the multidimensional nature of receptor activation and the differential sensitivities of assay systems (16, 17). GPCR agonists are typically tested in heterologous cell lines that express unphysiologically high receptor densities. These systems have spare receptors, and the resultant amplification of signaling facilitates high-sensitivity pharmacology but hampers translation of in vitro to in vivo pharmacology (17, 18). To determine unambiguously the in vitro pharmacologic properties of tirzepatide, we constructed HEK293 cell lines expressing defined levels of the human GIPR and GLP-1R (Supplemental Table 1; supplemental material available online with this article; https://doi.org/10.1172/jci.insight.140532DS1). As the Gα_s_/cAMP second messenger pathway is the principal insulinotropic signaling network activated by GIPR and GLP-1R (19), these low–receptor density assays represent systems with minimal signal amplification that can be used to understand the intrinsic pharmacology of GIPR and GLP-1R agonists.

**Figure 1:**
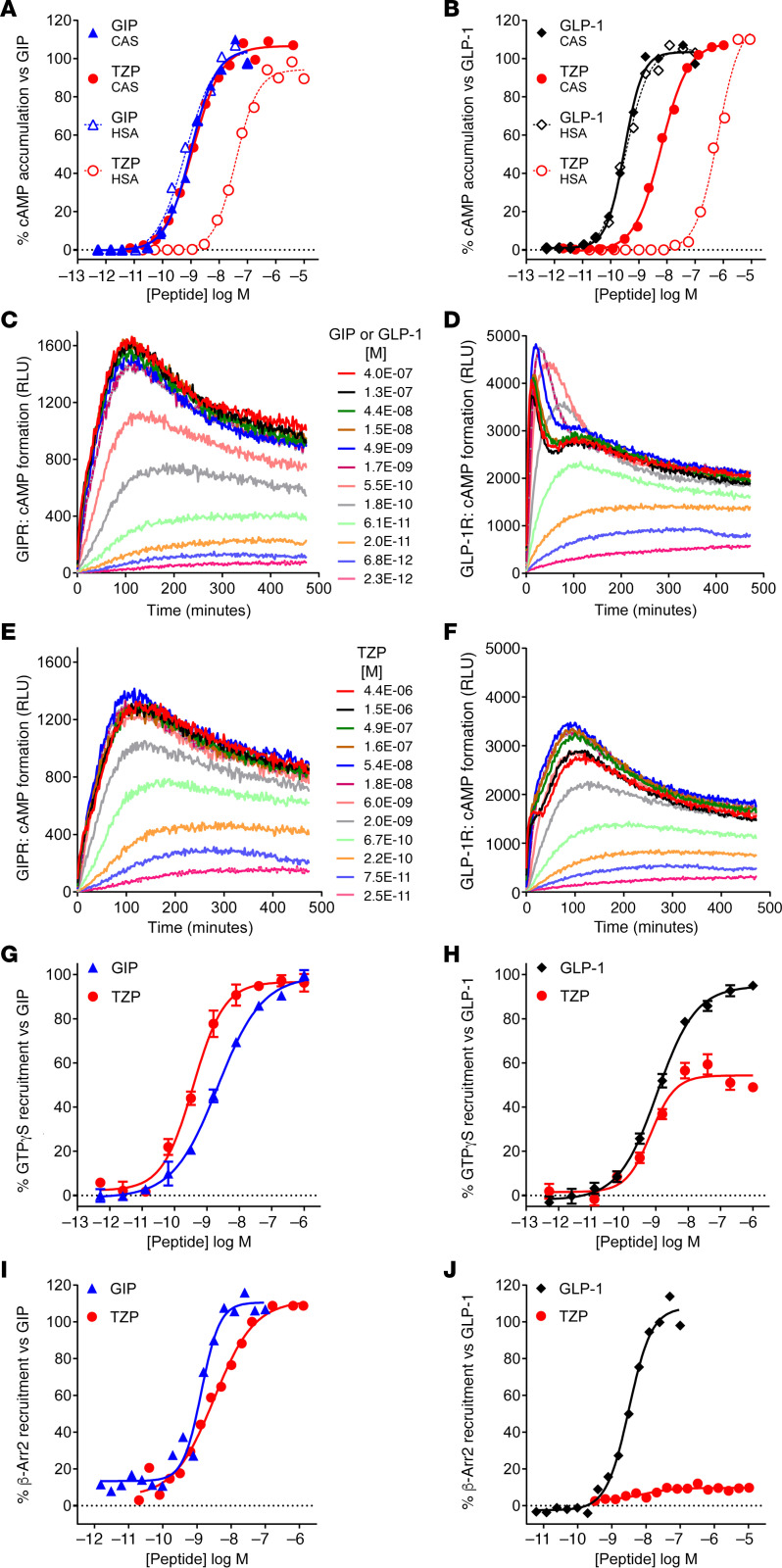
Tirzepatide (TZP) is an imbalanced agonist of the GIP and GLP-1 receptors and shows biased pharmacology at the GLP-1 receptor. (**A–F**) Intracellular cAMP accumulation was measured in low-density human GIPR- and GLP-1R–expressing HEK293 cells. (**A**) The intrinsic potency of TZP (*n* = 23) in the presence of casein (CAS) is equivalent to GIP(1-42) (*n* = 49). In the presence of human serum albumin (HSA), the potency of TZP right shifts 26-fold (*n* = 5), while GIP(1-42) is unaltered (*n* = 15). (**B**) The intrinsic potency of TZP (*n* = 22) is approximately 18-fold lower than GLP-1(7-36) (*n* = 57). In the presence of HSA, TZP right-shifts 81-fold (*n* = 5), while the potency of GLP-1(7-36) is unaltered (*n* = 24). (**C–F**) Agonist induced generation of cAMP was measured kinetically using a luminescence biosensor. Data are representative of 3 experiments. At the GIPR, GIP(1-42) (**C**), and TZP (**E**) have identical kinetic profiles. On the GLP-1R, native GLP-1(7-36) (**D**) has a complex profile with a bi-phasic kinetic response at high ligand concentrations, while TZP (**F**) is monophasic even at the highest tested concentrations. (**G** and **H**) Agonist-stimulated GTPγS binding of Gα_s_ in GIPR and GLP-1R in HEK293 cell membranes are presented as the mean ± SEM of 3 independent experiments. (**G**) On the GIPR, TZP is fully efficacious with an EC_50_ (SEM, *n*) of 0.379 nM (0.070, 3) versus GIP(1-42) of 1.43 nM (0.18, 27). (**H**) On the GLP-1R, TZP is a partial agonist 51% stimulation (5.2, 3) with an EC_50_ of 0.617 nM (0.190, 3) versus GLP-1(7-36) of 1.63 nM (0.21, 26). (**I** and **J**) The recruitment of ARRB2 to GIPR and GLP-1R in CHO-K1 cells. Representative data are presented. (**I**) The potency of TZP to recruit ARRB2 to GIPR is 2.34 nM (0.60, 7) and is comparable with GIP(1-42) of 1.58 nM (0.52, 6). (**J**) The potency of TZP to recruit ARRB2 to GLP-1R is difficult to determine due to low efficacy (*n* = 5), while GLP-1(7-36) is a full agonist with an EC_50_ of 3.26 nM (0.71, 14).

The acyl chain of tirzepatide contributes to albumin binding and half-life extension in vivo (12) (Supplemental Figure 1). Therefore, to determine the intrinsic pharmacology of tirzepatide relative to the native ligands GIP(1-42)NH_2_ (GIP) and GLP-1(7-36)NH_2_ (GLP-1), it was necessary to use albumin-free in vitro assays (Figure 1 and Supplemental Table 1). Tirzepatide showed a comparable affinity with GIP in competition binding and displayed equipotency compared with the native ligand in low–receptor density cAMP assays, along with full agonist activity on the GIPR (Figure 1A and Supplemental Table 1). By contrast, at the GLP-1R, tirzepatide demonstrated a 5-fold lower affinity in competition binding and 20-fold lower potency in cAMP accumulation (Figure 1B and Supplemental Table 1). A consistent 20-fold rightward shift in tirzepatide potency relative to GLP-1 was observed at all densities of GLP-1R expression (Supplemental Table 1). To investigate the biological impact of acylation on tirzepatide pharmacology, we measured the potency for cAMP accumulation in the presence of 1% (w/v) human serum albumin (HSA) to simulate the in vivo effect of albumin. A substantial decrease in potency for both GIPR and GLP-1R activation was observed in the presence of HSA, consistent with the formation of a nonsignaling HSA-tirzepatide complex (Figure 1, A and B). As anticipated, there was no effect of HSA on potency for the native ligands (Figure 1, A and B, and Table 1), while the GLP-1R agonist semaglutide (C18 di-acid conjugated) (20) was also substantially right-shifted (Table 1). We calculated the dissociation constant for tirzepatide interaction with HSA as 1.86 μM using modified Schild regression analysis and extrapolated this to determine the predicted free drug concentrations based on measured total values from clinical studies (Table 1). Based on the assumption that GLP-1R agonist pharmacology in the low–expression density cells best represent receptor affinity in vivo, the calculated free drug exposure values were used to generate predicted receptor occupancy (pRO) values (Table 1). Consistent with the imbalanced potency ratio of tirzepatide, the pRO estimates of engagement for the GIPR are higher than those for the GLP-1R (Table 1).

The kinetics of cAMP modulation by tirzepatide in the low-expression cell lines were investigated using a luminescence biosensor in the absence of phosphodiesterase inhibitors (21). Relative to GIP, tirzepatide showed a similar kinetic profile for cAMP production and fade at the GIPR (Figure 1, C and E). By contrast, GLP-1 displayed a complex profile for modulation of cAMP levels, with high ligand concentrations giving a biphasic response (Figure 1D). Distinct from GLP-1, tirzepatide exhibited a monophasic profile for GLP-1R–stimulated cAMP production (Figure 1F). To further understand the differential pharmacology of tirzepatide at the GLP-1R, agonist-induced GTPγS binding was measured in cell membranes (Figure 1, G and H). In this system, we observed that tirzepatide is a potent, partial agonist (51% efficacious) at the GLP-1R, while retaining full agonism at the GIPR.

Given the surprising divergent pharmacology for Gα_s_ activation and adenylyl cyclase modulation, we characterized other aspects of tirzepatide signal transduction, including non–G protein–mediated signaling pathways (22). Agonist-induced recruitment of β-arrestin2 (ARRB2) to the GIPR and GLP-1R was quantified using β-galactosidase–based enzyme fragment complementation (23). Tirzepatide displayed full agonism and close to equipotency with GIP for arrestin recruitment at the GIPR (Figure 1I). At the GLP-1R, however, tirzepatide exhibited a low efficacy (<10% E_max_), partial agonist profile for arrestin recruitment (Figure 1J). To exclude possible anomalous pharmacology by the artificial nature of the complementation assay, additional independent techniques were used and showed an equivalent pharmacological profile for tirzepatide recruitment of ARRB1 and ARRB2 at both the human and mouse GLP-1R (Supplemental Figure 2). These findings are generally consistent with results recently reported by Yuliantie et al. (24). In our studies, despite exhibiting low efficacy, the relative potency of tirzepatide for arrestin recruitment correlates well with binding affinity (Supplemental Table 1). Our data thus indicate that tirzepatide has a propensity toward partial agonism, and this ultimately reflects in signaling bias toward the cAMP pathway versus β-arrestin recruitment.

### Tirzepatide differentially induces internalization of the GIPR versus the GLP-1R.

**Figure 2:**
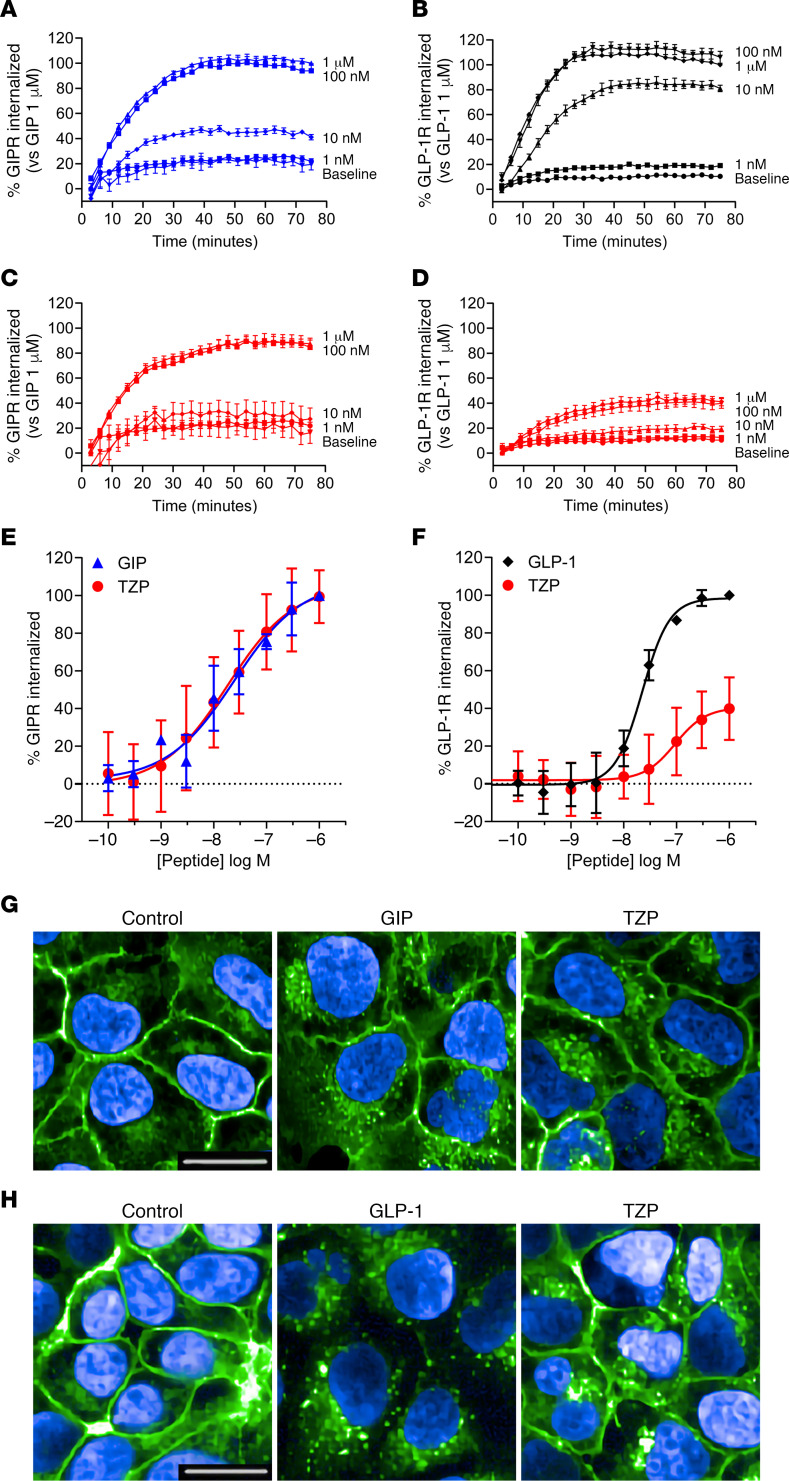
Tirzepatide (TZP) differentially induces internalization of the GIP and GLP-1 receptors. (**A–D**) The time course of internalization of GIPR (**A** and **C**) and GLP-1R (**B** and **D**) was assessed using changes in cell surface presentation of SNAP-tagged receptors in HEK293A cells. Receptor internalization induced by increasing concentrations of GIP(1-42) (**A**), GLP-1(7-36) (**B**), or tirzepatide (**C**, GIPR; **D**, GLP-1R) over time relative to the maximum signal for either GIP(1-42) (1 μM) or GLP-1(7-36) (1 μM) is shown. (**E–H**) Studies using receptors containing an N-terminal HA-epitope tag and a C-terminal EGFP fusion are presented. (**E**) Changes in surface GIPR 60 minutes after treatment with ligand were measured by anti-HA immunofluorescence. Data are normalized to 1 μM GIP(1-42) values. For GIPR, tirzepatide induced internalization with an EC_50_ (SEM, *n*) of 18.1 nM (5.7, 4), while GIP(1-42) displayed a potency of 18.2 nM (9.7, 4). (**G**) Representative confocal images of HA-GIPR-EGFP cells detecting EGFP fluorescence following treatment with vehicle, 100 nM GIP(1-42), or 100 nM tirzepatide. (**F**) Changes in surface GLP-1R 30 minutes after treatment with ligand detected by anti-HA immunofluorescence. Data are normalized to 1 μM GLP-1(7-36) values. For GLP-1R, tirzepatide was partially efficacious at 43.6% (7.9, 3) with an EC_50_ of 101.9 nM (29.8, 3), while GLP-1(7-36) showed a potency of 22.2 nM (1.86, 3). (**H**) Representative confocal images of HA–GLP-1R–EGFP cells detecting EGFP fluorescence following treatment with vehicle, 100 nM GLP-1(7-36), or 100 nM tirzepatide. Scale bars: 20 μm.

Agonist-dependent GPCR internalization is commonly mediated by arrestin recruitment of endosomal trafficking machinery (25) and is implicated in the differential pharmacology of class B GPCR agonists (26–28). We therefore quantified tirzepatide-induced receptor internalization using 3 independent techniques. Both GIP and tirzepatide induced internalization of N-terminally SNAP-tagged GIPR in a time and concentration dependent manner (Figure 2, A and C). The potencies and maximum internalization was comparable for both ligands. By contrast, there were clear differences between GLP-1– and tirzepatide-induced internalization of SNAP-tagged GLP-1R (Figure 2, B and D). Tirzepatide was less effective at internalizing GLP-1R relative to GLP-1 (<40% E_max_) (Figure 2D). To corroborate these findings, internalization was measured by detecting cell surface expression of the GIPR and GLP-1R utilizing an on-cell Western assay. HEK293 cells expressing GIPR with an N-terminal HA-epitope tag and a C-terminal EGFP fusion were used (29). Consistent with other reports, saturating amounts of GIP induced a low fraction of total GIPR internalization (35%) (28, 29). Plasma membrane GIPR was detected, and the potencies and extent of internalization by GIP and tirzepatide were found to be equivalent (Figure 2E). Identical studies were performed with the GLP-1R, and saturating GLP-1 induced a loss of the majority of plasma membrane receptors, in-line with other reported data (30). The ability of tirzepatide to induce internalization was much weaker, resulting in a maximum effect of only 40% of that observed with GLP-1 (Figure 2F). The low efficacy of tirzepatide to induce GLP-1R internalization is consistent with its limited ability to recruit β-arrestin (Figure 1J and Supplemental Figure 2). Finally, confocal imaging was utilized to visualize trafficking of these receptors in HEK293 cells expressing the HA and EGFP dual-tagged receptors. In response to treatment with GIP or tirzepatide, minimal changes in membrane-associated GIPR were observed (Figure 2G). Increased intracellular puncta were detected in the perinuclear region of GIPR cells treated with either ligand (Figure 2G), indicative of equivalent levels of agonist-induced receptor internalization (31). However, GLP-1 treatment induced nearly complete loss of annular cell surface staining for the GLP-1R (Figure 2H), and an increase in punctate localization of GLP-1R to the cytoplasmic/perinuclear region was observed. By contrast, treatment with tirzepatide resulted in a minimal reduction in cell surface labeling and only a small increase in punctate localization of receptor to the cytoplasmic/perinuclear region (Figure 2H). Collectively, these data illustrate tirzepatide, relative to native GLP-1, has low efficacy for internalization of the GLP-1R, while it is a full agonist for GIPR internalization. Together, these findings are reminiscent of profiles reported for an analogue of exendin-4 and a small molecule agonist, where both show reduced β-arrestin recruitment (26, 32) and the exenatide derivative has weak GLP-1R endocytosis (26).

### Lack of ARRB1 in pancreatic β cells enhances GLP-1R–activated insulin secretion.

**Figure 3:**
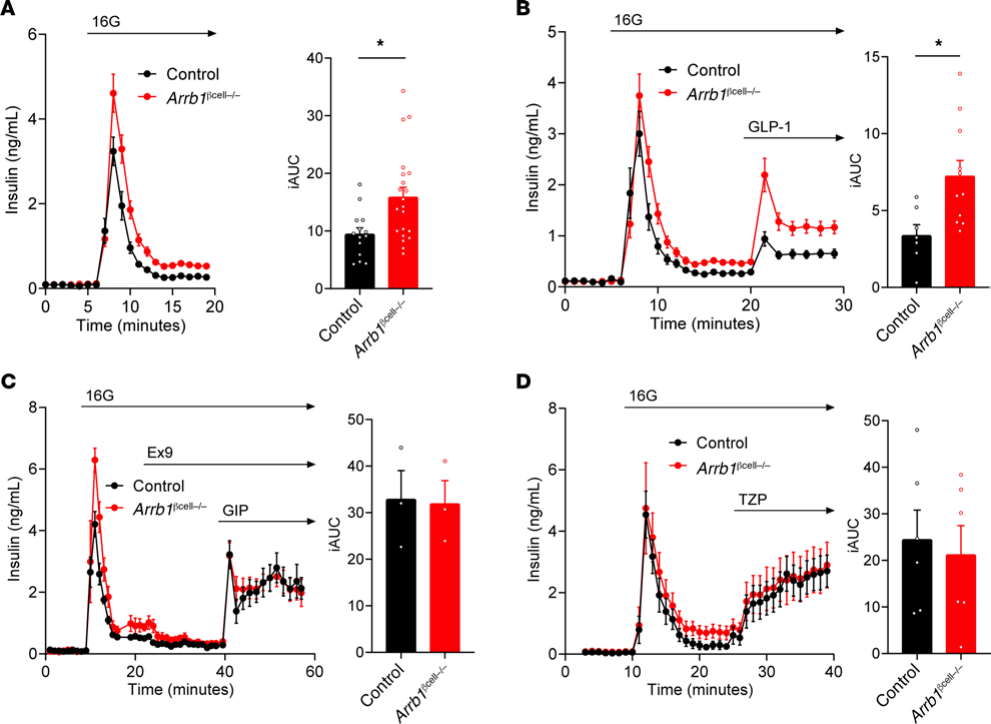
Deletion of β-arrestin1 in pancreatic β-cells increases GLP-1 receptor–activated insulin secretion. Islets from littermate controls and *Arrb1*^βcell–/–^** mice (male) were perifused ex vivo, and insulin secretion was measured in response to glucose (**A**), GLP-1 (**B**), GIP (**C**), or tirzepatide (TZP; **D**). Islets from *Arrb1*^βcell–/–^** mice secreted more insulin compared with control islets in response to 16 mM glucose (**A**) and 300 pM GLP-1 (**B**). By contrast, insulin secretion was not different between the sets of islets in response to either 3 nM GIP (**C**) or 1 nM tirzepatide (**D**). Exendin-4_(9–39)_ (Ex9; 1 μM) was used prior to GIP stimulation to normalize the elevated glucose stimulated insulin secretion. The integrated AUC (iAUC) was determined during the stimulation period: 6–19 minutes for 16 mM glucose (**A**), 20–29 minutes for GLP-1 (**B**), 40–58 minutes for GIP (**C**), and 24–39 minutes for tirzepatide (**D**). Each panel depicts results of a representative experiment from at least 2 independent experiments. *P<0.05, values are mean ± SEM. Statistical differences in iAUC values were determined by a 2-tailed student’s *t* test.

To determine the effects of biased GLP-1R signaling on function in primary cells, insulin secretion was measured in islets from WT mice and a line with β cell deletion of ARRB1 (*Arrb1^βcell–/–^*) using perifusion. In this system, addition of tirzepatide caused a concentration-dependent increase in glucose-stimulated insulin secretion (GSIS) in normal islets (Supplemental Figure 3A); the magnitude of this response was generally comparable with results with either GIP or GLP-1, although direct comparisons were not made. GSIS was elevated in *Arrb1^βcell–/–^* compared with control islets (Figure 3A), an effect that was abrogated by treatment with the GLP-1R antagonist exendin-4_(9–39)_ (Ex9; Figure 3C). Absence of *Arrb1^βcell–/–^* increased the insulin response to GLP-1 (Figure 3B), supporting a role for ARRB1 to restrain GLP-1R activity. However, this was not the case for GIP (Figure 3C and Supplemental Figure 3B) or tirzepatide (Figure 3D) stimulation, since insulin secretion was similar in WT and *Arrb1^βcell–/–^* islets. These findings are in keeping with the β-arrestin recruitment data in HEK293 cells (Figures 1J and Supplemental Figure 2). To determine whether tirzepatide activity at the GIPR could mask effects of β-arrestin at the GLP-1R, perifusions of normal islets (Supplemental Figure 3C) were repeated in the presence of a GIPR antagonist (33, 34). With GIPR activity blocked, tirzepatide was functionally a single receptor agonist in the perifusion system. Even so, the insulin secretory response to tirzepatide was the same in both genotypes (Supplemental Figure 3D), indicating that ARRB1 does not mute tirzepatide activity at the GLP-1R as it does with GLP-1. These findings in primary β-cells with a functional readout are compatible with the data obtained in heterologous cell systems and support a distinct profile of signaling properties for tirzepatide at the GLP-1R. The translational relevance of these findings is subject to investigation in a clinical study directly examining tirzepatide pharmacology (ClinicalTrials.gov, NCT03951753).

## Discussion

The analyses in this report provide a pharmacological basis for the efficacy of tirzepatide, and there are 3 key implications of the findings relevant to the broader class of multireceptor agonists. First, the pRO method presented here experimentally determines the fraction of unbound drug and then applies the total concentration of measured drug at steady state to correlate efficacious doses of tirzepatide with corresponding estimates of target engagement. This is an underappreciated aspect of incretin receptor biology, as there is often a lack of understanding regarding target engagement. For drug discovery, this is especially challenging for acylated peptides because it is difficult to determine circulating concentrations of free drug. We found that the pRO values for GLP-1R are substantially lower than for GIPR (Table 1). This is consistent with the imbalanced design of tirzepatide favoring GIP over GLP-1R affinity. Furthermore, insights are offered when comparing the pRO of tirzepatide versus semaglutide for the GLP-1R, as tirzepatide requires lower GLP-1R engagement than semaglutide to deliver equivalent glucose and weight control efficacy (9, 35) — i.e., comparison of 5 mg tirzepatide (pRO 3%) with 1 mg semaglutide (pRO 6%) (Table 1). Similarly, dose escalation of tirzepatide, that may match (10 mg, pRO 7%) or exceed (15 mg, pRO 10%) the occupancy of GLP-1R by 1 mg semaglutide, is accompanied by superior efficacy compared with efficacy observed with this selective GLP-1R agonist (9, 35). Although the field awaits head-to-head study of these agents (ClinicalTrials.gov, NCT03987919), the estimates of comparative GLP-1R occupancy support a role of GIPR agonism in tirzepatide for enhancing the efficacy of its GLP-1R pharmacology (11). More broadly, estimating target engagement using the approach taken here may help designing other multireceptor agonists, especially those incorporating glucagon signaling where threshold limits in certain tissues are likely.

We recognize that calculating pRO values has caveats (see Methods) and propose its use mainly for generating hypotheses and as a tool to probe mechanistic biology. For perspective, though, when examining the pRO values of other ligands, we observe that GLP-1R occupancy is also relatively low (about 10%). Based on established data, this makes sense in terms of the in vivo sensitivity of the GLP-1R system. For instance, exendin-4 efficacy for rodents in acute glucose challenge experiments occurs at a pRO of 13% (36) (in vivo EC_50_ = 40 pM, in vitro EC_50_ = 260 pM). In humans, active GLP-1 and GIP released following nutrient ingestion provides pRO in the general range of 1%–4% for GLP-1R and 4%–20% for GIPR (Table 1) (37, 38). This is likely due to spare receptors, in that the threshold of occupancy for a maximal physiological response of a full agonist requires considerably less than 100% occupancy. It can also be hypothesized that the GLP-1/GLP-1R system is inherently more sensitive than the GIP/GIPR system, as both pairings have similar interaction affinities yet the biological levels of GIP are higher (e.g., after feeding) (39, 40). To further validate the pRO method, it will be crucial to extend the approach to measuring target engagement using advanced tracer approaches that ultimately may offer insights into pRO across tissues. There is progress in this area with the recent description of both GLP-1 and glucagon receptor PET ligands (41, 42).

A second important implication of this report is in the design of poly-pharmacological drugs possessing GLP-1 activity. Here, GLP-1R potency must be carefully considered because dose escalation of selective GLP-1R agonists induces undesirable gastrointestinal effects (13). Such effects have not been described for GIP, even in an infusion study where the concentration of GIP reached the estimated free drug levels of the higher tirzepatide doses (Table 1) (40); in turn, this is not surprising, given the reported antiemetic effects of GIP analogues (43). Thus, a dual agonist with imbalanced potency favoring GIP over GLP-1 is hypothesized to allow dosing schemes that maximize GIP effects while simultaneously achieving a GLP-1 response that is efficacious and tolerable. Tirzepatide has the same affinity and potency properties as native GIP but is comparatively weaker at the GLP-1R. Although it is unclear whether the degree of imbalance for tirzepatide is fully optimal, its efficacy in early trials is more robust than that reported for NNC00902746, a balanced dual agonist (Supplemental Figure 4) (44, 45).

Lastly, and arguably the most intriguing consideration of the work herein, is the consideration of biased agonism and its influence in optimizing the observed metabolic efficacy of GLP-1R agonists. In this regard, the observation that tirzepatide selectively engages cAMP signaling over β-arrestin recruitment at the GLP-1R may be of fundamental importance that beneficially impacts GLP-1R trafficking, thus augmenting cellular response. Indeed, others have reported that GLP-1R agonists possessing similar signaling bias are more effective than matched, unbiased agonists at controlling glucose and body weight in mice (26, 46). The apparent benefit of biased GLP-1R agonism, combined with strong GIPR-induced glucose lowering shown in mechanistic studies of *Glp1r-*null mice (12), may provide tirzepatide the ability to improve metabolic control across a spectrum of patients where responsiveness to either incretin varies. Together, tirzepatide displays pharmacology unique to any multiincretin agonist evaluated to date in the clinic. It may be described as an “imbalanced” agonist both in terms of its stronger affinity and potency at the GIPR versus the GLP-1R but also owing to its biased agonism of the GLP-1R compared with its pleiotropic activity at the GIPR. This mix of properties establishes an important benchmark and thereby merits investigating biased pharmacology for other emerging agonists. For tirzepatide, the full realization of its integrated dual receptor engagement and signaling properties awaits readout of ongoing phase 3 registration and clinical mechanism of action trials.

## Methods

GLP-1(7-36)NH_2_, GIP(1-42)NH_2_, NNC00902746, semaglutide, and tirzepatide were either synthesized and purified to > 95% purity at Eli Lilly and Company or obtained from Caslo. The GIPR antagonist is the anti-GIPR antibody encoded by the sequences 3161/3162-2.63.1 previously reported (33, 34). All other reagents were of the highest purity available from Thermo Fisher Scientific, unless otherwise described.

### cDNA constructs.

Cell line generation and in vitro pharmacology experiments were performed with pcDNA3.1 mammalian expression constructs encoding WT or modified human GIPR (NP_000155), human GLP-1R (NP_002053), or mouse GLP-1R (NP_067307). Human GLP-1R–HaloTag and NanoLuc-ARRB2 fusion constructs were used for the measurement of β-arrestin recruitment with NanoBRET technology (47). For NanoLuc enzyme complementation, mouse or human GLP-1R–LgBiT and ARRB1-SmBiT or ARRB2-SmBiT fusion constructs were generated (48). For receptor internalization studies, DNA encoding human GIPR and GLP-1R with N-terminal 3X-HA epitope tags and C-terminal EGFP tags was constructed in pcDNA3.1. For the real-time internalization assay, the N-terminally SNAP-tagged GIPR was synthesized by Cisbio and inserted into pcDNA3.1. Mammalian expression DNA encoding an N-terminally SNAP-tagged GLP-1R was received as a gift from Hans Bräuner-Osborne (University of Copenhagen, Denmark) (49).

### Generation of clonal cell lines and determination of receptor density.

We constructed HEK293 cell lines with defined GIPR and GLP-1R levels that we classify as low (about 2000 receptors/cell), medium (about 10,000 receptors/cell), and high (about 100,000 receptors/cells) receptor densities (Supplemental Table 1). HEK293T cells were obtained from ATCC (CRL-3216) and cultured using standard methods and media as described (50). Stable cell lines were generated by the transient transfection of 150 cm^2^ flasks of cells with pCDNA3.1 expression vectors using 15 μg DNA complexed with 60 μL Fugene-6 (Promega), according to manufacturer directions. Cells were clonally selected by antibiotic resistance to G418 (400-800 μg/mL; Cellgro) or Zeocin (100 μg/mL; Invitrogen). Individual cell clones were generated by single cell dilution plating. Cell clones were expanded and screened by assaying the potency of cAMP accumulation in response to native ligands, and then the receptor density of selected clones was determined by radioligand binding. Receptor number and pharmacology were unambiguously determined using respective native ligands GIP(1-42) and GLP-1(7-36) and iodinated analogues. The respective affinities of GIP(1-42) and GLP-1(7-36) as determined by radioligand binding were 200 pM and 700 pM, consistent with the literature (51, 52). In line with the expected pharmacology, we observed that GIP(1-42) and GLP-1(7-36) potency for cAMP accumulation was dependent on receptor density and ranged from low pM at high expression levels to potency values approaching nM at low expression levels (Supplemental Table 1) (18). The number of receptors per cell was calculated based on radioligand binding B_max_ (maximum specific binding) values and the experimentally determined number of cells/mg of cell membrane protein.

### [^125^I]GLP-1(7-36)NH_2_ and [^125^I]GIP(1-42)OH binding.

Membranes from HEK293 cells expressing the cloned human GLP-1R or human GIPR were prepared as described previously (50). Receptor expression density was determined using homologous competition, and *K_i_* values were determined using competition binding. Incubations were performed in a total volume of 200 μL in a 96-well plate (Corning). [^125^I]GLP-1(7-36)NH_2_ or [^125^I]GIP(1-42)OH (2200 Ci/mmol, ≥ 95 % purity, final assay concentrations ~0.12–0.20 nM, PerkinElmer) in assay buffer (2.5 mM MgCl_2_ [MilliporeSigma], 1.0 mM CaCl_2_ [MilliporeSigma], 0.1% w/v fraction V fatty acid free bovine serum albumin [82-002-4, MilliporeSigma], 0.1% w/v bacitracin [11805, Affymetrix] in 25 mM HEPES [Thermo Fisher Scientific], pH 7.4, final concentrations) was added to peptide in assay buffer (concentration-response curves in DMSO, 3-fold acoustic direct dilution [Echo555, Labcyte], final DMSO concentration in the assay was 0.96%). Assay buffer (100 μL) containing GLP-1R or GIPR membranes that had been preincubated at room temperature with WGA-PVT SPA beads (PerkinElmer) for 2 hours was added. The amount of membrane protein and WGA-SPA bead depended on the expression density and was 0.5–1.0 μg protein with 0.25 mg bead for the high-, 1.5–7.5 μg protein with 0.25–0.5 mg bead for the medium-, and 5.0–20 μg protein with 0.25–0.75 mg bead for the low-expressing cell lines. The plates were covered with sealing tape, mixed, and incubated for an additional 18 hours at room temperature. The plates were centrifuged at ~200*g* for 5 minutes at room temperature. Bound ligand was determined using a MicroBeta Trilux Scintillation Counter (PerkinElmer). Competition binding with human GLP-1(7-36)NH_2_, GIP(1-42), tirzepatide, and semaglutide was performed essentially as described for homologous competition except that the assay buffer was 1.0 mM MgCl_2_, 2.5 mM CaCl_2_, 0.003% w/v Tween-20, 0.1% w/v bacitracin in 25 mM HEPES, pH 7.4, final concentrations with one Complete EDTA free protease inhibitor tablet added per 50 mL of buffer. Using GraphPad Prism 7 software, B_max_ values for [^125^I]GLP-1(7-36)NH_2_ or [^125^I]GIP(1-42) binding to GLP-1R and GIPR membranes were determined by nonlinear regression analysis using the amount bound versus the concentration of competing homologous peptide added. The B_max_ was used to calculate the number of receptors per cell. For competing peptides, K_i_ values were determined by nonlinear regression analysis using the amount of [^125^I]GLP-1(7-36)NH_2_ or [^125^I]GIP(1-42) bound versus the concentration of peptide added.

### cAMP accumulation assays.

Clonal HEK293 cells stably expressing either untagged human GIPR or human GLP-1R at various levels of receptor density were used for measurement of cAMP accumulation using the Gs Dynamic Assay and homogenous time-resolved fluorescence (62AM4PEJ, PerkinElmer). Corning 3570 white microtiter plates were used to collect 10 μL assay medium (DMEM, 31053, [Thermo Fisher Scientific], 2 mM Glutamax [35050, Thermo Fisher Scientific], 20 mM HEPES, 0.1% w/v bovine casein [4765, MilliporeSigma], and 250 μM isobutylmethylxanthine [IBMX] phosphodiesterase inhibitor) from a Multidrop Combi dispenser (Thermo Fisher Scientific). For assays containing albumin from human serum, assay medium contained an additional 1% w/v fatty acid free HSA (A3782, MilliporeSigma). To create concentration response curves, theses plates were used as destination vessels for 100 nL direct dilution using an Echo 555 acoustic liquid handler and DMSO as a ligand diluent. Treatment plates were warmed to 37°C prior to cell addition. Vials of assay-ready cells were rapidly thawed from cryopreservation on the day of the assay. The freezing medium was removed, and cells were resuspended in the appropriate assay medium lacking IBMX and placed at 37°C for 60 minutes. Following recovery, an equal volume of cell suspension was added to the prewarmed treatment plates and allowed to accumulate cAMP at 37°C for an additional 30 minutes. The cells were lysed by sequential addition of d2-labeled cAMP competitor conjugate and cryptate-conjugated detection antibody for 1 hour at room temperature. Time-resolved fluorescence was quantitated with a Pherastar FSX multilabel reader (BMG Labtech). Fluorescence data were analyzed by the ratio method, calibrated to external standards in a parallel processed plate, and reported as percent activation compared with vehicle minimum and native peptide maximum control wells. Normalized percent values were fit to the 4-parameter logistic model in Genedata Screener or GraphPad Prism 7 software.

### Kinetic cAMP assays.

Kinetic cAMP assays were performed in low-density GIPR and GLP-1R cell clones transfected with the Glosensor 22F vector (Promega) (53). Adherent cells were transiently transfected with Glosensor at 7.5 μg DNA/30 μL Fugene-6 per 150 cm^2^ flask, according to manufacturer’s directions. After 48 hours, cells were removed from flasks using enzyme-free dissociation buffer (13151014, Thermo Fisher Scientific), followed by trituration, brief centrifugation (500*g*, 5 minutes, room temperature) and filtration (40 μm cell strainer, Thermo Fisher Scientific). Cell viability was quantified with a ViCell (Beckman Coulter) and was typically > 85%. Cells were resuspended at ambient temperature in CO_2_-free media (18045088, Thermo Fisher Scientific) containing 0.1 %(v/v) casein (C4765, MilliporeSigma) and 2%(w/v) Glosensor detection reagent (Promega). A total of 18,000 cells per well was plated in 180 μL in solid-bottom, white 96-well plates (3917, Corning). Luminescence was quantified in kinetic mode using a temperature-controlled (22°C) EnVision (PerkinElmer) using standard luminescence settings. Cells were equilibrated for 5–20 minutes, and then 20 μL of 10× ligand was added and a luminescence time course was collected. Ligand (10×) was titrated by manual serial dilution in DMSO followed by step-down into assay buffer.

### GTPγS recruitment.

The potency of ligands to stimulate receptor-dependent elevation of GTP**γ**S-bound Gα_s_ subunit was determined using membrane preparations previously described from the low–receptor density human GIPR and GLP-1R clonal cell lines. GTP recruitment reactions contained 2.5 μg of membrane in 20 mM HEPES (pH 7.4), 10 mM NaCl, 5 mM MgCl_2_, 0.004% w/v saponin (47036, MilliporeSigma), 0.1% bacitracin (11702, MilliporeSigma), and 500 pM [^35^S]guanosine 5′-(gamma-thio)triphosphate (PerkinElmer). Formulated ligands were diluted to 100× working stocks in DMSO prior to being diluted in aqueous assay buffer to a final concentration of 1×. Reactions were incubated for 30 minutes at room temperature in white, clear-bottom microtiter plates. To terminate reactions and to allow for antibody capture, 0.2% NP40 detergent and moderate vortexing were used to solubilize receptor/G protein complexes. During detergent addition, a custom rabbit anti-Gα_s_/olf polyclonal antibody created like sc-383 (Santa Cruz Biotechnology Inc.), used to mimic its properties, and 0.25 mg of anti–rabbit IgG polyvinyltoluene scintillant beads (RPNQ0016, PerkinElmer) were also added to the mixture. The detection mixtures were developed for 30 minutes and centrifuged at 80*g* for 10 minutes to collect SPA beads at the bottom of microtiter wells, and photons were counted in normal mode for 1 minute/well using a MicroBeta TriLux instrument (PerkinElmer). Experimental observations were generated on separate days. Percent of the maximal response was calculated using control wells containing saturating amounts of native GIP or GLP-1. Relative EC_50_ value was derived by nonlinear regression analysis using the percent response versus the concentration of ligand and fitted to a 4‑parameter logistic equation using GraphPad Prism 7 software.

### β-Arrestin recruitment assays.

ARRB2 recruitment to human GIPR and GLP-1R was quantified in CHO-K1 PathHunter cells expressing C-terminally fused ProLink1 receptors and Enzyme Acceptor fused ARRB2 from DiscoverX (Eurofins). Corning 3570 microtiter plates were used to collect 10 μL assay medium (McCoy’s 5A medium Hyclone SH30200, 1× penicillin-streptomycin [pen/strep; Hyclone, SV30010], 0.1% bovine casein [MilleporeSigma, C4765]) and 100 nL ligand via acoustic direct dilution, as mentioned above, prior to prewarming at 37°C. Cell vials were thawed and added at equal volume in respective assay medium as previously stated. Recruitment of ARRB2 to activated receptors occurred at 37°C for 90 minutes, followed by cell lysis by the addition of 10 μL detection mixture containing β-galactosidase 93-0001 substrate to quantitate functional enzyme fragment complementation. Chemiluminescent signal was developed for 60 minutes at room temperature and quantified using an EnVision plate reader. Raw data were normalized to percent activation compared with control wells, and the curves were fit to the 4-parameter logistic model in Genedata Screener. As an alternative approach to measure β-arrestin recruitment, bioluminescent resonance energy transfer (BRET) was performed. The NanoBRET method system (Promega) was used to measure the interaction of NanoLuc-ARRB1/2 and human GLP-1R-HaloTag proteins (47). Freestyle HEK cells (R79007, Thermo Fisher Scientific) were transfected in antibiotic-free media using Fugene-6 complexed in optiMEM (Thermo Fisher Scientific). Following 48-hour shaking, cells were pelleted and resuspended in assay buffer (1× PBS, 0.1% w/v bovine casein). BRET was performed with the Nano-Glo substrate (N1662) as donor and NanoBRET 618 ligand as acceptor. Emission was measured at 460 nm (donor) and 610 nm (acceptor) wavelengths. The BRET ratio was calculated as acceptor emission divided by donor emission. Similar methods were used to measure β-arrestin recruitment to the mouse GLP-1R ortholog using NanoLuc enzyme complementation system. Transient transfection, as above, was used to deliver DNA encoding mouse GLP-1R–LgBiT and ARRB1/2-SmBiT fusion proteins. Agonist-stimulated protein-to-protein interaction of 2 fusion molecules were detected with Nano-Glo substrate and standard luminescent detection. Data analysis was using GraphPad Prism 7 software.

### Real-time internalization assay.

HEK293 cells were seeded in white, 384-well plates the day before transfection at a density of 20,000 cells/well. The cells were transfected with the calcium phosphate precipitation method for SNAP-GIPR and with Lipofectamine 2000 (Invitrogen) for SNAP–GLP-1R. The following day, the media was removed and the tagged receptors were labeled with 100 nM Tag-Lite SNAP-Lumi4-Tb (donor, Cisbio), in OptiMEM for 75 minutes at 37°C. Afterward, the cells were washed with internalization buffer (HBBS supplemented with 1 mM CaCl_2_, 2.5 mM MgCl_2_, 20 mM HEPES, and 0.1% Pluronic F-68, pH 7.4) followed by addition of 100 μM preheated fluorescein-O’-acetic acid (acceptor, Sigma-Aldrich). The plate was placed in a 37°C incubator for 5 minutes prior to ligand addition to adjust the temperature. Then, the cells were stimulated with 37°C preheated ligand, and internalization was measured every 3 minutes for 60 minutes at 37°C by an EnVision plate reader. Data were normalized to maximum concentrations of GLP-1 or GIP (100%) and no ligand (0%) and plotted using GraphPad Prism 7 software.

### On-cell Western assay.

HEK293 cells stably expressing HA-GIPR-EFGP or HA–GLP-1R–EFGP clones were plated into poly-D-lysine–coated 96-well microplates and cultured until cells reached 80%–90% confluency. On the day of assay, growth media was removed, and cells were rinsed once with prewarmed starvation media (growth media without serum or antibiotics, supplemented with 0.1% casein) and equilibrated with fresh media for 1 hour at 37°C, 5% CO_2_. Concentration response curves of GLP-1, GIP, and tirzepatide were prepared in prewarmed starvation media, added to cells for designated times, and incubated at 37°C. At the end of the study, media was removed, and cells were placed on ice and fixed with Prefer fixative (Anatech) for 10 minutes. Fixative was removed, and cells were washed in PBS and blocked with Odyssey blocking buffer (Licor) for 1 hour. Cells were incubated with anti-HA/DyLight800 antibody (1:700) (Rockland Immunochemicals, 600-445-384) for 1 hour followed by washes with PBS-T. Plates were scanned using a Licor Clx scanner with the 800 nm channel laser to capture fluorescence signal in each well. Data were normalized to maximum concentrations of GLP-1 or GIP (100%) and no ligand (0%) and analyzed by nonlinear regression (sigmoidal concentration-response) and plotted using GraphPad Prism 7 software.

### Confocal imaging.

Cells were plated into poly-D-lysine–coated 96-well cyclic olefin imaging microplates (PerkinElmer). Internalization assays were performed as described above. At the end of the study, cells were fixed with 4% paraformaldehyde for 10 minutes, washed in PBS, and blocked in PBS plus 2% BSA for 45 minutes. Internalization was performed as described above, and cells were then fixed with 4% paraformaldehyde and washed with PBS. Nuclear staining was performed by incubation with Hoechst 33342 stain (0.5 μg/mL) in PBS for 5 minutes. Cells were imaged using the Opera Phenix system (PerkinElmer) by spinning-disk confocal fluorescence imaging with a water-immersion 20× objective and appropriate channels for GFP (excitation, 499 nm; fluorescence, 520 nm) and Hoechst (excitation, 350 nm; fluorescence, 461 nm).

### Animals.

*Arrb1^βcell–/–^* mice were generated by crossing *Arrb1^fl/fl^* mice (54) with MIP-Cre^ERT^ mice (55). Control (*Arrb1^fl/fl^*) and *Arrb1^βcell–/–^* mice were treated with tamoxifen at 8 weeks of age (4 mg daily, orally, for 5 consecutive days) and then sacrificed for islet isolation at 15–18 weeks of age. Breeders were maintained on standard breeder chow (Lab Diet, 5058), all other mice received standard rodent chow (Research Diets, 5053).

### Pancreatic islet isolation.

Islets were isolated by inflating the pancreas with collagenase type V (0.8 mg/mL) in HBSS injected retrograde through the pancreatic duct, as previously described (56). Briefly, digestion occurred at 37°C and was stopped with application of ice-cold RMPI (Thermo Fisher Scientific, 11875119, 2 mM L-glutamine, 0.25% BSA). Islets were separated from pancreatic tissue using a histopaque gradient and allowed to recover in RMPI (11.1 mM glucose, 10% FBS [Thermo Fisher Scientific, 10437028], 1% pen/strep) overnight before experiments were performed.

### Pancreatic islet perifusion.

For islet perifusion, 75 islets were handpicked and loaded into 0.275 mL chambers containing KRPH (140 mM NaCl, 4.7 mM KCl, 1.5 mM CaCl_2_, 1 mM NaH_2_PO_4_, 1 mM MgSO_4_, 5 mM HEPES, 2 mM NaHCO_3_, 1% fatty acid-free BSA) in 2.7 mM glucose. Upon initiation of the perifusion experiment, KRPH with 2.7 mM glucose + 1% BSA was perifused at a rate of 200 μL/min for 24 minutes, followed by KRPH with 2.7 mM glucose +0.1% BSA, to provide an equilibration period for the islets. Following equilibration, experimental conditions were applied, and perifusate was collected each minute. GLP-1 and GIP were diluted in KRPH prior to experiment. Perifusate insulin concentrations were measured with AlphaLISA (PerkinElmer). The following concentrations were used for each reagent: GLP-1, 300 pM; GIP, 3 nM; tirzepatide, 1 nM; GIPR antagonist, 300 nM; exendin-4_(9–39)_ (Ex9), 1 μM.

### Determination of ligand affinity for albumin using albumin-shift assays.

Methods to quantify and predict the effect of albumin binding on biological activity in enzyme assays, receptor binding assays, and cell-based assays exist, but they are not currently widely employed (57–59). The general approach involves performing a bioassay in an albumin-free system and quantifying the effect of systematic titrations of albumin into the system. Pharmacologic models can then be used to calculate the albumin affinity of the test compound. Theoretical considerations of this methodology are described (57–59). Briefly, consider the equilibrium system:

 (Equation 1)
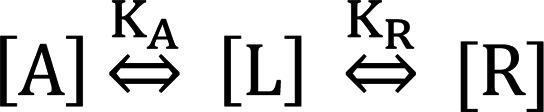


Where R is receptor (e.g., GIPR or GLP-1R), A is albumin, and L is a ligand that can bind to both the receptor and albumin (e.g., acylated GIPR/GLP-1R agonist). A direct mathematical solution for the concentrations of various components given initial conditions and equilibrium constants does exist but is not straightforward (57). Moreover, this method is impractical for in vivo or cell assays, where the receptor concentration is typically unknown. Various standard mathematical expressions for the system can be derived:

 (Equation 2)
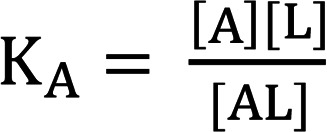


 (Equation 3)
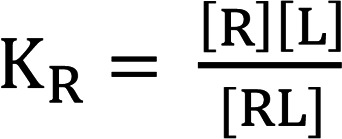


 (Equation 4)
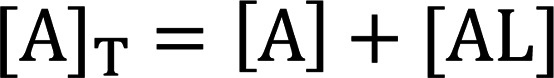


 (Equation 5)
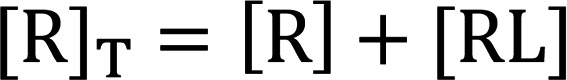


 (Equation 6)
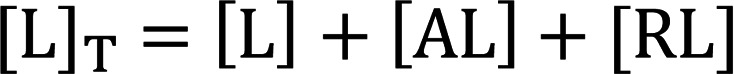


Equations 2 and 3 describe the equilibrium constants for the system, and equations 4, 5, and 6 express the conservation of mass ([A]_T_, [R]_T_, and [L]_T_ are total concentrations). These can be used to derive a Schild type relationship, as described (59, 60). Given that experiments will be conducted in the presence of different concentrations of albumin, ligand quantities can be considered L_T1_ for [A] = 0 and L_T2_ for [A] > 0 that give an equiactive response.

Thus, in the presence of albumin, [A] > 0.

 (Equation 7)
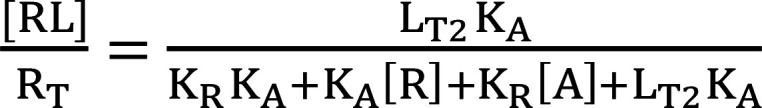


In the absence of albumin, [A] = 0, then

 (Equation 8)
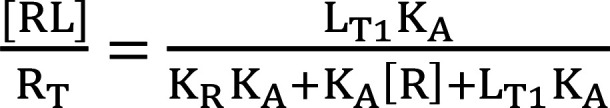


 (Equation 9)
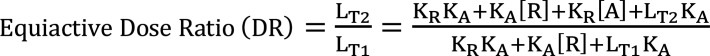


 (Equation 10)
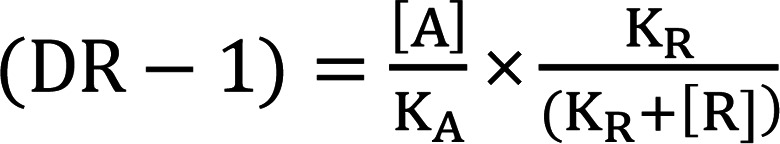


We note the R species–containing term in Equation 10 will approximate 1 for values of R << K_R_ . For all reasonably constructed in vitro and in vivo assays K_R_/(K_R_ + [R]) ≈ 1. For example, in the present study, [R] in GIPR and GLP-1R low–expression density assays are 300 fM and 200 fM, respectively. Therefore, for the most potent peptides used in this study, [R] is approximately 1000-fold below the K_R_ values (Supplemental Table 1). Thus, the equation can be simplified and converted to the logarithmic form:

 (Equation 11)
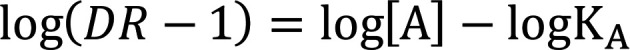


This can be used to fit a titration series and determine an accurate value of K_A_ by Schild regression or analogously determine an estimate of K_A_ from a single concentration shift:

 (Equation 12)
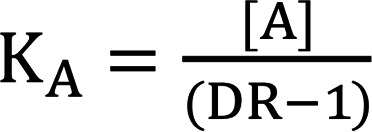


We determined K_A_ values for a number of acylated GPCR agonist ligands using the albumin-shift approach. The cell assay buffer contained 0.1% casein to prevent nonspecific ligand adsorption, and we varied the assay albumin concentration. Matched pairs of acylated and unacylated ligands demonstrated the expected pharmacology with large rightward potency shifts for acylated peptides, and the potency of unacylated peptides was independent of albumin concentrations. The K_A_ values calculated for known acylated peptides matched reasonably well with literature values for unbound fraction (e.g., we determined semaglutide to have a K_A_ of 460 nM and a predicted unbound fraction of 0.00072 at 640 μM albumin, consistent with the highly albumin-bound nature of this molecule) (20, 61).

### Calculation of predicted unbound drug concentration in plasma.

For calculating free-drug levels, we assume that the impact of receptors is negligible based on relative concentrations at steady state ([R]_T_ (low pM levels at maximum) << [L]_T_ (nM levels for peptide ligands) << [A]_T_ ≈ 600 μM). Thus, one can consider the system at equilibrium:

 (Equation 13)
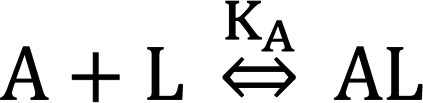


The total concentrations of species, [A]T and [L]T are given by:

 (Equation 14)
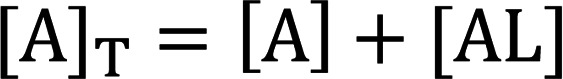


 (Equation 15)
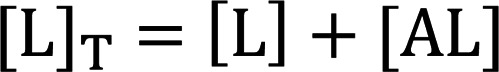


The concentration of the AL complex is given by:

 (Equation 16)
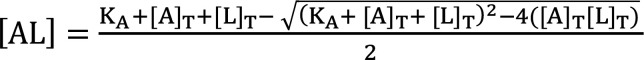


Therefore, the concentration of free L is given by:

 (Equation 17)
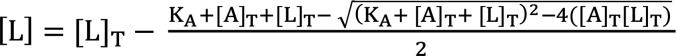


As [L]_T_ and [A]_T_ and K_A_ are measured quantities, [L] can be determined by calculation. We used 640 μM as the [A]_T_ value, as it is the midpoint of the albumin reference interval (62). In parallel, we confirmed results using the cubic solution method of Blakeley (57).

### Calculation of receptor occupancy.

To calculate a theoretical value for receptor occupancy based on unbound plasma concentration, we use the Hill-Langmuir equation:

 (Equation 18)
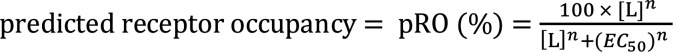


where [L] is predicted unbound plasma ligand concentration, EC_50_ or K_D_ from target engagement assay, and *n* = Hill slope for the biological response. This relationship assumes the biological assay to determine target engagement is amplification free and accurately reflects receptor affinity and biology of the receptor such that the EC_50_/K_D_ accurately captures the free ligand concentration required for activation in an unamplified system. It also assumes the assays used to obtain the EC_50_/K_D_ are binding protein free and have negligible nonspecific binding. For GIPR and GLP-1R, Hill coefficients for the low-density cAMP assays approximated to 1. For calculations in Table 1, we used low-density cAMP assays, as these are believed to best represent target engagement for several reasons. These assays show differential pharmacology between molecules that binding assays do not, and they likely capture some aspects of pharmacology that equilibrium radioligand binding assays do not (binding kinetics, desensitization, subcellular distribution of compounds). In vivo, cAMP is the principal insulinotropic mediator; thus, using a cell-based method to determine the influence of acylation on potency and for predicting pRO is logical. This methodology also assumes that the measurement of [L]_T_ is accurate. This is likely to be the case, as clinical trial assays for [L]_T_ are typically robust and well validated. We note that this analysis, while founded in analytical pharmacology (63), is primarily designed to help contextualize existing data and generate new hypotheses to be tested.

### Statistics.

All data are presented as mean ± SEM. Statistical analyses were performed using GraphPad Prism 7 software. A 2-tailed Student’s *t* test or 2-way ANOVA was performed, depending on the experimental design, with a Bonferroni post hoc analysis. *P* < 0.05 was used to determine statistical differences.

### Study approval.

All procedures for the use of the mice followed protocols approved by the IACUC of Duke University.

## Author contributions

FSW, ADS, MBNG, DBW, TMS, WJCVDV, CS, GRC, PJE, MMR, and KWS designed and/or performed the pharmacological studies. FSW, DBW, and SU performed the receptor occupancy analysis. JDD, MEC, DAD, and JEC designed and/or performed the ex vivo insulin secretion studies. FSW, ADS, MBNG, DBW, TMS, JDD, MEC, CS, GRC, PJE, MMR, JEC, and KWS performed data analysis. FSW, ADS, TMS, JJH, DAD, MPC, MMR, JEC, and KWS wrote and/or edited the manuscript. FSW, MMR, JEC, and KWS supervised the project.

## Supplementary Material

Supplemental data

## Figures and Tables

**Table 1:**
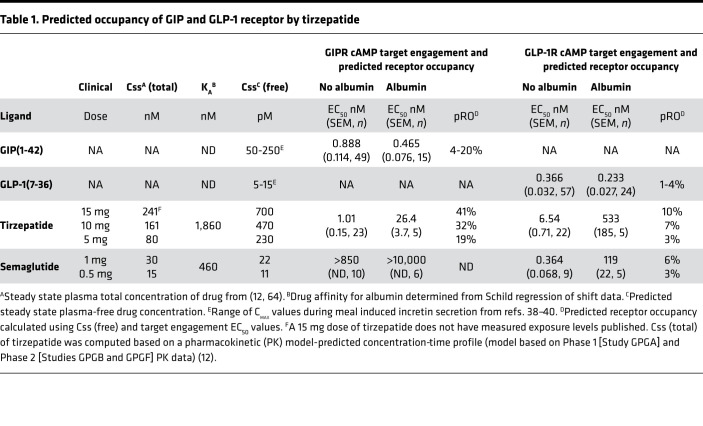
Predicted occupancy of GIP and GLP-1 receptor by tirzepatide

## Version 1 07/30/2020

In-Press Preview

## Version 2 09/03/2020

Electronic publication
