# Supplementary material for: Tirzepatide is an imbalanced and biased dual GIP and GLP-1 receptor agonist
Source: JCI Insight. 2020 Sep 3;5(17):e140532. doi: 10.1172/jci.insight.140532 (PMC7526454; doi:10.1172/jci.insight.140532)
Supplement: Supplemental data [file jciinsight-5-140532-s251.pdf]

Supplemental Information – Table & Figures

Tirzepatide is an Imbalanced and Biased Dual GIP/GLP-1 Receptor Agonist

	[^{125}I]GIP Binding		GIPR Low Density cAMP			GIPR Medium Density cAMP			GIPR High Density cAMP		
Ligand	K_i nM [A] (SEM, n)	K_i [B] Ratio	Rec/Cell [C] (SEM, n)	EC_{50} nM (SEM, n)	EC_{50} [D] Ratio	Rec/Cell [C] (SEM, n)	EC_{50} nM (SEM, n)	EC_{50} [D] Ratio	Rec/Cell [C] (SEM, n)	EC_{50} nM (SEM, n)	EC_{50} [D] Ratio
**GIP**	0.203 (0.033, 12)	1.00		0.888 (0.114, 49)	1.00		0.0461 (0.0084, 12)	1.00		0.00224 (0.00040, 6)	1.00
**Tzp**	0.163 (0.029, 6)	1.25	1,700 (500, 5)	1.01 (0.15, 23)	0.88	6,400 (900, 6)	0.0536 (0.0157, 8)	0.86	86,000 (29,000, 6)	0.00196 (0.00019, 2)	1.14
**Sema**	>500 (ND, 2)	ND		>850 (ND, 10)	ND		>100 (ND, 2)	ND		80.3 (16.1, 2)	0.00

	[^{125}I]GLP-1 Binding		GLP-1R Low Density cAMP			GLP-1R Medium Density cAMP			GLP-1R High Density cAMP		
Ligand	K_i nM [A] (SEM, n)	K_i [B] Ratio	Rec/Cell [C] (SEM, n)	EC_{50} nM (SEM, n)	EC_{50} [D] Ratio	Rec/Cell [C] (SEM, n)	EC_{50} nM (SEM, n)	EC_{50} [D] Ratio	Rec/Cell [C] (SEM, n)	EC_{50} nM (SEM, n)	EC_{50} [D] Ratio
**GLP-1**	0.655 (0.055, 12)	1.00		0.366 (0.032, 57)	1.00		0.0721 (0.0061, 24)	1.00		0.00624 (0.00056, 14)	1.00
**Tzp**	2.94 (0.33, 6)	0.22	1,400 (200, 5)	6.54 (0.71, 22)	0.06	7,800 (1,300, 6)	1.38 (0.19, 11)	0.05	76,000 (10,000, 7)	0.132 (0.006, 9)	0.05
**Sema**	1.10 (0.28, 5)	0.60		0.364 (0.068, 9)	1.01		0.0571 (0.0117, 4)	1.26		0.00671 (0.00042, 3)	0.93

Supplemental Table 1. *In Vitro* Activity of Tirzepatide in Transgenic Cell Lines with Varying Receptor Densities. [A] Affinities were calculated from competition binding data using the Cheng-Prussoff correction (*Biochem Pharmacol.* 1973;22(23):3099-108). [B] Calculated by ratio of native ligand K_i to test ligand K_i. [C] Receptors per cell calculated by radioligand binding B_{MAX} determinations. [D] Calculated by ratio of native ligand EC_{50} to test ligand EC_{50}.

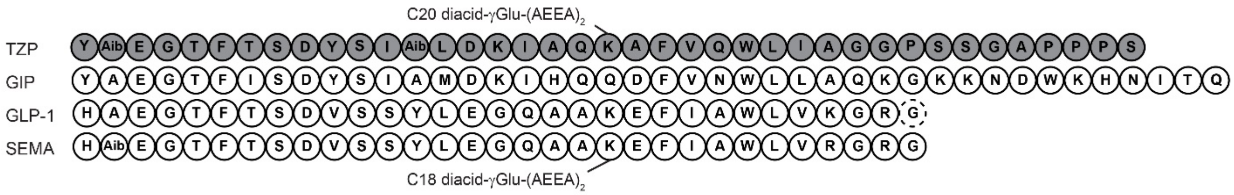

Supplemental Figure 1. Multiple Sequence Alignment of Ligands. Depicted is an amino acid sequence comparison of tirzepatide (TZP), human GIP(1-42), human GLP-1(7-36), and semaglutide (SEMA). For tirzepatide and semaglutide, the locations of the engineered acyl moieties are indicated. The dotted circle at the end of the GLP-1 sequence represents the terminal glycine residue that is present in the naturally occurring GLP-1(7-37) variant.

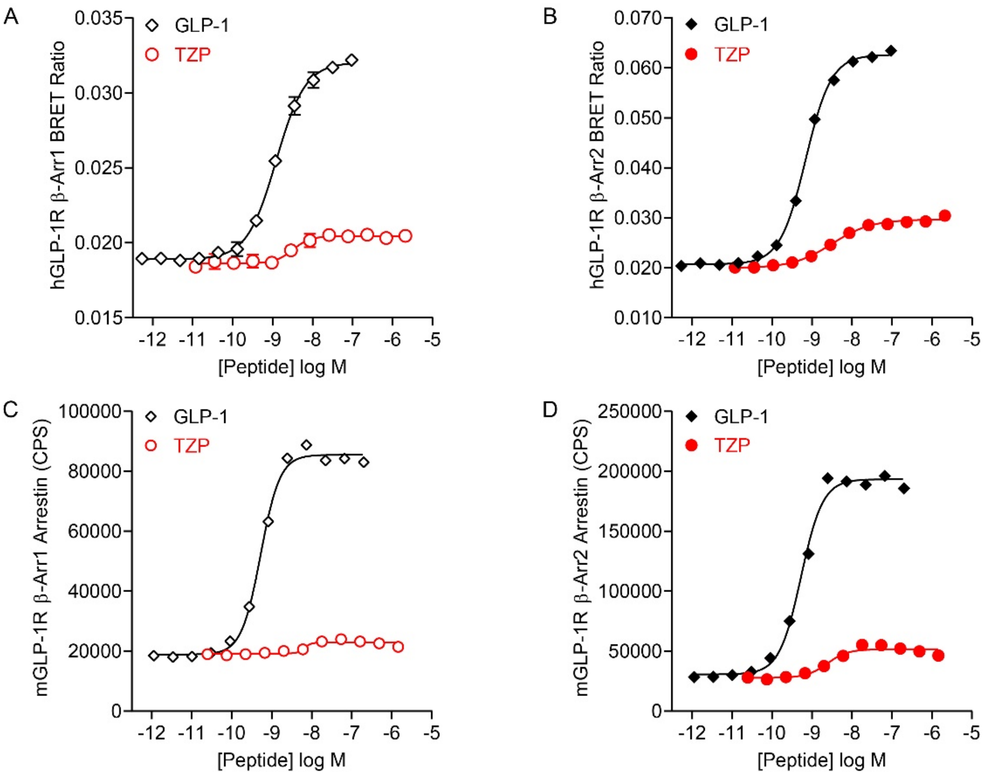

Supplemental Figure 2. β-Arrestin1 and β-Arrestin2 Recruitment to the GLP-1 Receptor. Arrestin recruitment was measured using bioluminescence resonance energy transfer (BRET) and NanoLuciferase complementation (NanoLuc). (**A**) Recruitment of β-Arr1 to human GLP-1R using BRET. EC_{50} values and efficacy relative to GLP-1: GLP-1 (1.3 nM, 100%) Tzp (3.1 nM, 12%) (**B**) Recruitment of β-Arr2 to human GLP-1R using BRET. EC_{50} values and efficacy relative to: GLP-1 (0.7 nM, 100%), Tzp (3.2 nM, 23%) (**C**) Recruitment of β-Arr1 to mouse GLP-1R using NanoLuc complementation. EC_{50} values and efficacy relative to GLP-1: (0.53 nM, 100%), Tzp (6.6 nM, 6%) (**D**) Recruitment of β-Arr2 to mouse GLP-1R using NanoLuc complementation. EC_{50} values and efficacy relative to GLP-1: GLP-1 (0.53 nM, 100%), Tzp (2.4 nM, 15%).

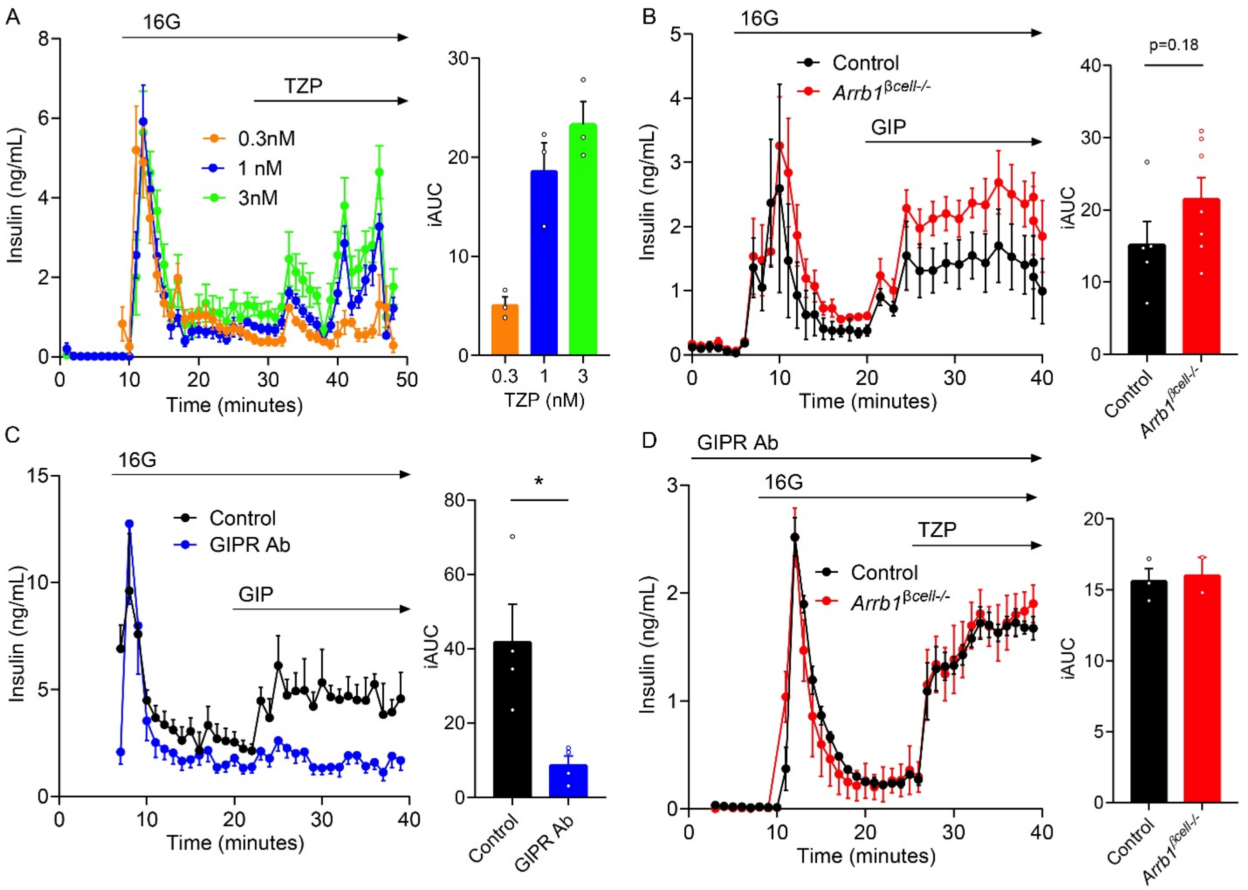

Supplemental Figure 3. Insulin Secretion in Perifused Islets. (**A**) Tirzepatide (TZP) enhances glucose stimulated insulin secretion from normal islets (wild-type male) in a concentration-dependent manner. Integrated area under the curve (iAUC) is determined from 25-39 minutes. (**B**) Insulin secretion in response to GIP is similar in control and *Arrb1*$^{\beta cell\text{-}/\text{-}}$ islets. iAUC is determined from 20-40 minutes. (**C**) GIP stimulated insulin secretion is prevented by a GIPR antagonist. iAUC is determined from 21-39 minutes. (**D**) Insulin secretion in response to TZP is similar in control and *Arrb1*$^{\beta cell\text{-}/\text{-}}$ islets in the presence of the GIPR antagonist. iAUC is determined from 25-39 minutes. Each panel depicts results of a representative experiment from at least 2 independent experiments. * - *P*<0.05, values are mean ± SEM. Statistical differences in iAUC values were determined by a 2-tailed student's *t* test.

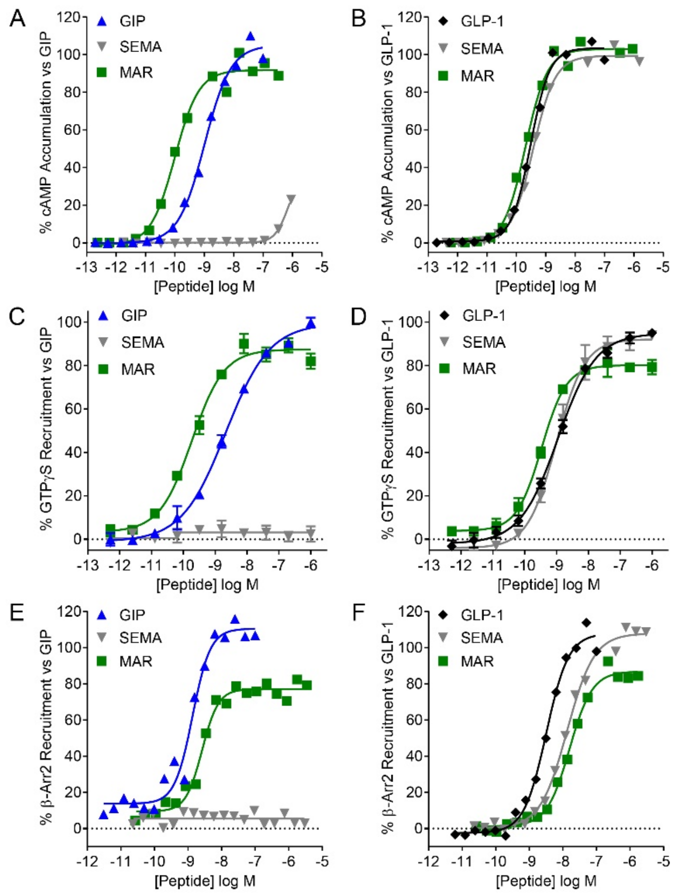

Supplemental Figure 4. In Vitro Pharmacology of Comparator Ligands. The intrinsic activity of semaglutide (SEMA) and NNC00902746 (MAR/MAR709/RG7697) for intracellular cAMP accumulation (**A, B**), agonist stimulated GTPγS binding of $G\alpha_s$ (**C, D**), and recruitment of β-Arr2 (ARRB2) (**E, F**) in comparison to native peptide as previously shown in Figure 1 and Table 1. Graphs are representative curves for summarized data and number of independent experiments, n, mentioned below. (**A**) The potency (SEM, n) of GIP(1-42) for cAMP accumulation on GIPR is 0.888 nM (0.114, 49). SEMA: >1,000 nM at n=10. MAR: 0.130 nM (0.017, 7). (**B**) The potency of GLP-1(7-36) for cAMP accumulation on GLP-1R is 0.366 nM (0.032, 57). SEMA: 0.364 nM (0.068, 9). MAR: 0.248 nM (0.035, 5). (**C**) The potency of GIP(1-42) for GTP recruitment for GIPR is 1.43 nM (0.18, 27). SEMA: >1,000 nM at n=2. MAR: 0.182 nM (0.028, 3). (**D**) The potency of GLP-1(7-36) for GTP recruitment for GLP-1R is 1.63 nM (0.21, 26). SEMA: 1.01 nM (0.13, 3). MAR: 0.333 nM (0.045, 3). (**E**) The potency of GIP(1-42) for ARRB2 recruitment to GIPR is 1.58 nM (0.52, 6). SEMA: >10,000 nM at n=3. MAR: 2.01 nM (0.31, 3) and is trending toward partial agonism at 79.4% (2.9, 3) compared to native 118% (11, 6). (**F**) The potency of GLP-1(7-36) for ARRB2 recruitment to GLP-1R is 3.26 nM (0.71, 14). SEMA: 16.8 nM (11.9, n=4). MAR: 7.19 nM (3.23, 5).
